# Lack of evolutionary adjustment to ambient temperature in highly specialized cave beetles

**DOI:** 10.1186/s12862-015-0288-2

**Published:** 2015-02-04

**Authors:** Valeria Rizzo, David Sánchez-Fernández, Javier Fresneda, Alexandra Cieslak, Ignacio Ribera

**Affiliations:** Institute of Evolutionary Biology (CSIC-Universitat Pompeu Fabra), Passeig Maritim de la Barceloneta 37–49, 08003 Barcelona, Spain; Museu de Ciències Naturals (Zoologia), Barcelona, Spain

**Keywords:** Acclimation, Adaptation, Pyrenees, Thermal tolerance, Subterranean environment

## Abstract

**Background:**

A key question in evolutionary biology is the relationship between species traits and their habitats. Caves offer an ideal model to test the adjustment of species to their surrounding temperature, as they provide homogeneous and simple environments. We compared two species living under different thermal conditions within a lineage of Pyrenean beetles highly modified for the subterranean life since the Miocene. One, *Troglocharinus fonti*, is found in caves at 4-11°C in the ancestral Pyrenean range. The second, *T. ferreri*, inhabits the coastal area of Catalonia since the early Pliocene, and lives at 14-16°C.

**Results:**

We found no differences in their short term upper thermal limit (ca. 50°C), similar to that of most organisms, or their lower thermal limit (ca. -2.5°C), higher than for most temperate insects and suggesting the absence of cryoprotectants. In longer term tests (7 days) survival between 6-20°C was almost 100% for both species plus two outgroups of the same lineage, but all four died between 23-25°C, without significant differences between them.

**Conclusions:**

Our results suggest that species in this lineage have lost some of the thermoregulatory mechanisms common in temperate insects, as their inferred default tolerance range is larger than the thermal variation experienced through their whole evolutionary history.

**Electronic supplementary material:**

The online version of this article (doi:10.1186/s12862-015-0288-2) contains supplementary material, which is available to authorized users.

## Background

One of the main questions in evolutionary biology is to link the phenotype of an organism to the environment in which it lives. This link is usually masked by the complexity of both the environmental factors that may influence an organism and its response to these factors [1,2], posing a major challenge to the understanding of the origin of phenotypic traits. Under natural conditions species are exposed to continuously changing and interacting climatic factors [3], to which they can adjust by behavioural plasticity, change of microhabitat or by migration [2,4].

The deep subterranean environment seems an ideal scenario to study the evolutionary adjustment of the species to their environments, as many of these confounding factors are completely absent from it. In the deepest parts of the caves and their surrounding network of fissures the conditions of the habitat are extremely constant and homogeneous, with a permanent darkness and nearly constant temperature and humidity [5,6]. Subterranean organisms have a very limited range of options to behaviourally exploit local climatic heterogeneity –as there is virtually none–, and their general lack of mobility [5,6] also reduces the possibility of migration when conditions become unfavourable. In addition, the general scarcity of resources imposes stringent requirements on the species living in this environment, resulting in simple, low diverse communities [7,8].

Caves have a homogeneous and constant temperature through the year, approximately equal to the average annual temperature of the surface [5,9], but for any given lineage of subterranean organisms this temperature may vary both geographically (including altitudinal changes) and temporally (past climatic change). It could be expected that given the harsh conditions and the stability of the environment species should have fine-tuned their thermal tolerance to the temperatures of their habitats [10-12]. For example, in a similarly constant and homogeneous environment, the cold Antarctic waters, the most stenotherm species cannot survive an increase of 2-5°C above the temperature they currently experience (below 2°C) [13]. Cave organisms are thus an ideal model to test the long term evolutionary adjustment of organisms to the temperature of their habitats [14], although there is very limited information available on their comparative thermal tolerances (see Discussion).

The Pyrenean chain in the Iberian peninsula is one of the world hotspots of subterranean diversity [15,16]. Two monophyletic radiations of beetles are particularly rich, with hundreds of narrow endemic species: Trechini [17,18] (Carabidae) and Leptodirini [19,20] (Leiodidae). Within the Pyrenean Leptodirini, there was a single colonisation of the subterranean environment in the Early Miocene, leading to a radiation of more than 140 subterranean species distributed through the chain [20]. Only in one occasion these species expanded their range outside the Pyrenees, with the colonisation of coastal areas of Catalonia during the early Pliocene by a member of the genus *Troglocharinus* that subsequently diversified in isolated karstic areas [21]. The caves occupied by the species in the coastal area are on average 5°C warmer than those occupied by the species of the ancestral range in the Pyrenees (average temperature of the caves in the coast 14.2 ± 1.0°C; in the Pyrenees 8.9 ± 1.9°C), offering a unique opportunity to study the long term evolutionary adjustment of the species to their surrounding temperatures and the possible persistence of regulatory or acclimation mechanisms.

**Figure 1:**
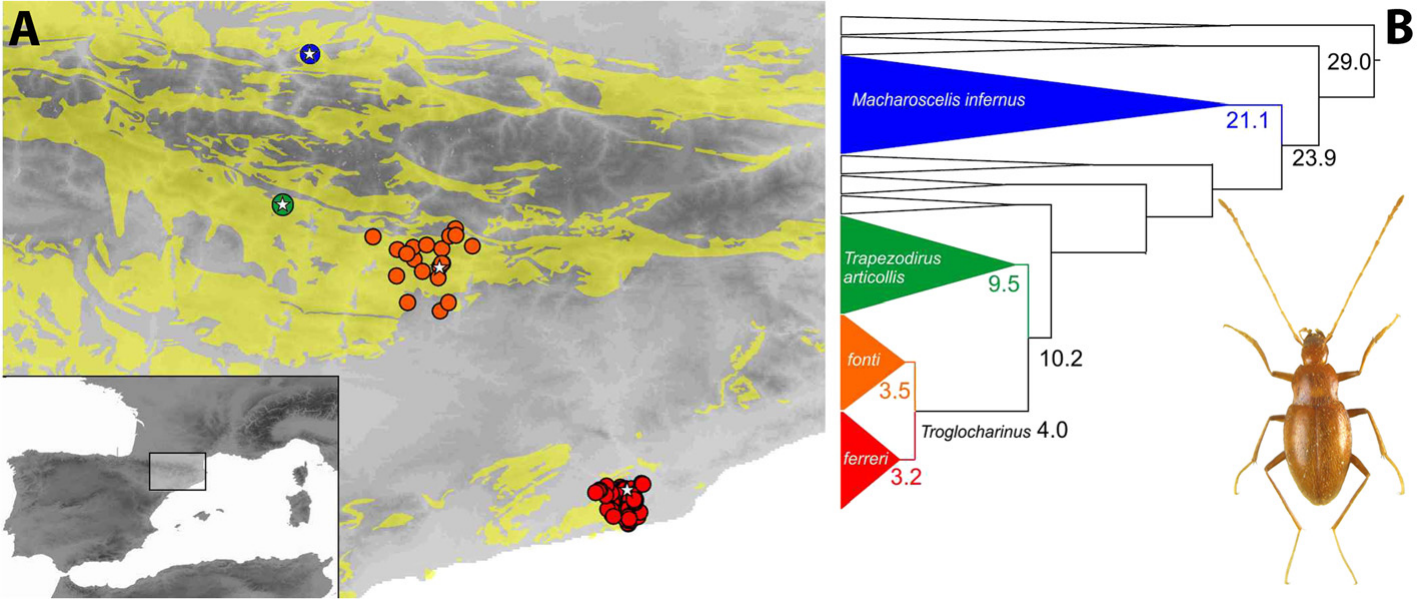
**Study system. A)** Geographical distribution of the species used in the experiments and **B)** phylogenetic relationships among them (modified from ref. [20]). Yellow background areas in the map, distribution of the limestone. Coloured circles, caves known to have the species; with a star, the cave from which the studied specimens were collected (*M. infernus* and *T. arcticollis* are only known from a reduced number of caves in close proximity, and their ranges are represented by a single circle). Numbers in the nodes, millions of years before present. Photo habitus, *Troglocharinus ferreri*.

In this work we test the thermal tolerance of two species of *Troglocharinus*, one from the ancestral area in the Pyrenees (*T. fonti* Jeannel) currently living in caves at ca. 4-11°C, and another from the coast (*T. ferreri* Reitter), found in caves between 14-16°C. We compare their short term upper (UTL) and lower critical thermal limits (LTL) as well as their longer term survival at a range of temperatures encompassing those they experience under natural conditions, or were likely to have experienced in their evolutionary history (as estimated from palaeoclimatic data). In addition to the two species of *Troglocharinus*, for the long term experiments we also studied two related Pyrenean species, one (*Trapezodirus arcticollis* Jeannel) within the same highly derived subterranean lineage of *Troglocharinus*, and another (*Macharoscelis infernus* Dieck) more distantly related (with a common ancestor ca. 24 Ma [20], Figure 1).

Using an experimental approach we aim to study the evolutionary adjustment of cave species to their surrounding temperatures by testing 1) if there is any significant difference in the short term thermal limits between the species living in the coast (*T. ferreri*) and the species living in the Pyrenees (*T. fonti*); 2) if there is any difference among the studied species in their longer term (up to 7 days) thermal tolerance at a range of subcritical temperatures; and 3) if there is any sign of acclimation to changing temperatures within their tolerance ranges.

The combination of an experimental approach with data on the current and past temperatures allows a better understanding of the long term evolutionary dynamics of the thermal biology of subterranean species, and its potential role in their geographical expansion and diversification. The knowledge of their thermal tolerances should also be of interest for the conservation prospects of these species, given their limited possibilities to track suitable conditions when confronting global change.

## Methods

### Short term experiments

For the short term thermal tolerance experiments we used two species of the genus *Troglocharinus*, one from the warmer coastal area (*T. ferreri*) and another from the ancestral geographic range within the Pyrenees (*T. fonti*) [21]. Specimens of *T. ferreri* were collected by hand in Cova de Coll Verdaguer (Garraf massif), with a temperature range of 14-16°C (Figure 1; Table 1) using baits and food traps set 24 h in advance. Specimens were placed in a portable fridge at ca. 14°C and 85% RH, with substratum from the cave and moss from the entrance to retain humidity, and kept and transported in the same conditions to the place where the experiments were conducted (University of Murcia, Spain).

**Table 1:** **Caves from which the studied specimens were collected**

**Species**	**Cave**	**Lat.**	**Long.**	**Range T current**	**Range T LGM**	**Range T LIG**
*T. ferreri*	Cova de Coll Verdaguer, Vallirana	41.393	1.911	13.9-16.2	9.4-11.7	13.9-16.3
*T. fonti*	Cova d’Ormini, Montanisell	42.207	1.226	4.2-10.9	(−0.6)-6.2	4.30-10.9
*T. articollis*	Cova de St Salvador, Bonansa	42.437	0.653	6.9	2.1	7.0
*M. infernus*	Gouffre du Béguet, Juzet d’Izaut	42.987	0.751	8.7-10.1	5.5-6.0	8.8-10.2

Data include geographical coordinates (see Figure 1), range of temperatures in which the species is currently found, estimated range of temperatures of the Last Glacial Maximum (LGM; 21,000 YBP), and range of temperatures of the Last Interglacial (LIG; 120,000-140,000 YBP).

Specimens of *T. fonti* were collected by hand in Cova d’Ormini (Pyrenees), at a temperature of 6-8°C (Figure 1; Table 1). Specimens were handled and stored in the same conditions as those used for *T. ferreri*, with the only exception of the temperature in the portable fridge, set to ca. 7°C.

To obtain an estimate of the average current temperature of the caves and associated subterranean environment in which the species are found we used the mean annual temperature at a 0.08 degree spatial resolution from WORLDCLIM, version 1.3 (www.worldclim.org [22]). The deep subterranean environment is known to have a constant temperature approximately equal to the average annual temperature of the surface [5,9]. To estimate the temperature during the Last Glacial Maximum (LGM; 21,000 YBP) we used a simulation of the general circulation model (GCM) from the Community Climate System Model (CCSM, http://www2.cesm.ucar.edu/ [23]). The original GCM data were downloaded from the PMIP2 website (https://pmip2.lsce.ipsl.fr/). For the Last Interglacial (LIG; 120,000-140,000 YBP) we used the data provided by [24], available at www.worldclim.org (Table 1).

For the experiments we used a FLIR SC305 infrared camera (FLIR Systems, Portland, US), with a resolution of 320x240 pixels and sensitivity <0.05°C at 30°C. The camera was connected to a computer using the ThermaCam Researcher Professional 2.10 software (FLIR Systems). Beetles were video taped in a climatic chamber BINDER MK53 (BINDER, Tuttlingen, Germany; range −50° to 180°C, ±0.3°C). During the experiments, specimens were fixed on their elytra with paper glue or bee wax.

**Figure 2:**
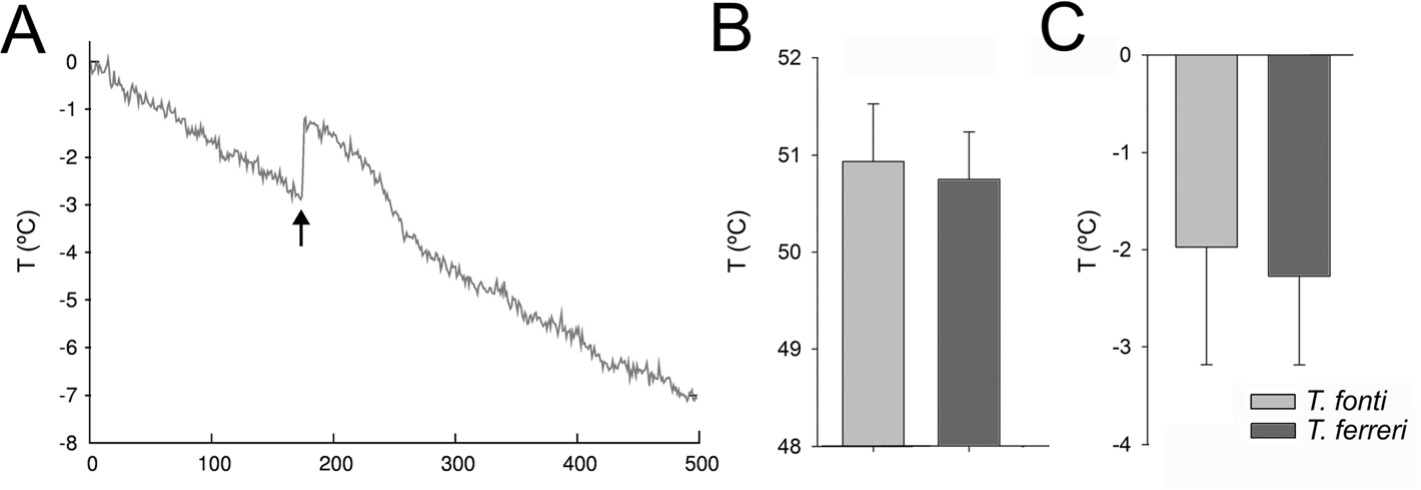
**Results of the short term experiments for the Pyrenean (** ***T. fonti*** **) and coastal (** ***T. ferreri*** **) species of** ***Troglocharinus.*** **A)** Example graph of the lower thermal limit (LTL) experiment. The arrow marks the point at which the specimen froze (i.e. the supercooling point), with the sudden liberation of latent (or crystallization) heat, considered to be the LTL. **B)** Upper thermal limit (UTL). **C)** LTL. Columns, average temperature at which specimens died, with standard deviation.

Experiments to determine the lower thermal limit (LTL) started at a temperature close to the range found in the caves of origin, with a decreasing ramping temperature of 1°C/min and an initial RH of 60-70%. Body temperature was continually recorded, with data logging at intervals of ca. 1". The temperature considered as the LTL was the supercooling point [25,26], that is, the point at which a sudden increase of body temperature was recorded, indicating the release of latent heat due to freezing (Figure 2A). We do not have evidence of freezing tolerance in any of the studied species, neither from our experiments nor from published data. Experiments were continued until −20°C.

For the upper thermal limit (UTL) we set up an increasing ramping program with the same general conditions as before. Upper thermal limit (i.e. the lethal temperature) was considered to be the point at which specimens stopped moving for more than a minute, as recorded by a video camera and observed through a visor by two different observers (e.g. [27]). Experiments were stopped at 60°C. We did not attempt to measure the critical thermal maximum (CTmax) as with glued specimens it was difficult to estimate the point at which they loose control of their movements. In the long term experiments it was also not possible to determine the CTmax (see below), the UTL being thus the only comparable measure between the two types of experiment.

For *Troglocharinus fonti* (the Pyrenean species) we did two replicates of 12 specimens for each the UTL and the LTL. For *T. ferreri* (the coastal species), and due to the limited availability of specimens, we did a single experiment of 12 specimens each for UTL and LTL.

### Long term experiments

To have an estimation of thermal tolerances at more ecologically relevant conditions we set up a series of experiments using survival at longer times and temperature increases at slower rates. We used the same two species as before (the coastal *Troglocharinus ferreri* and the Pyrenean *T. fonti*), plus two outgroups of the same lineage to establish the polarity of any potential change between the two *Troglocharinus* species. The species used as outgroups were *Trapezodirus arcticollis* and *Macharoscelis infernus*, both from the Pyrenees (see Figure 1 and Table 1 for the location and conditions of the caves; and ref. [20] for the phylogenetic relationships and divergence times between the four species).

Specimens were collected by hand in the caves and transferred to the laboratory under controlled conditions, with substratum from the cave and some moss to keep humidity. In the laboratory, specimens were acclimated and stored previous to the experiments at the approximate temperature of the cave (see Table 1) in a Liebherr WTes 4177 cabinet (Bulle, Switzerland), which can establish a continuous gradient of temperature between 5-18°C, in closed plastic containers (30x21x6.5 cm) with moss and substratum from the caves (the “stock”). Specimens were kept in these conditions, considered to be closer to the natural conditions in the cave, without being disturbed until used for the experiments. For the experiments, specimens were placed in smaller plastic boxes (13x8x5.5 cm) with a 0.5 cm white plaster substratum, ca. 10 volcanic stones of a diameter of 1–3 cm saturated with distilled water, and white tissue paper also saturated in distilled water. Specimens in the experimental boxes were fed *ad libitum* with freshly frozen *Drosophila melanogaster*. Some of the control specimens were kept in the same experimental boxes for more than nine months after the end of the experiments, indicating that the observed mortality was mostly due to the temperature treatments and not to stressing conditions or a reduced longevity (which in any case would have affected both control and treatment specimens equally).

For the experiments, the box with the specimens to be treated (usually replicas of 10 individuals) was placed in a Nüve TK120 (Ankara, Turkey) climatic test cabinet (temperature range −10°C/+60°C, relative humidity, RH, range 20-95%). Controls were kept in the cabinet at the approximate temperature of the cave and RH close to saturation. The actual temperature and RH, or temperature only, inside the experimental boxes were recorded every 30" during the experiments with data loggers Testo (Lenzkirch, Germany) 176H1 (T ± 0.2°C, RH ±0.1%) or Testo 176 T4 (T ± 0.5°C, with Cu-C termopars) (Additional file 1: Table S1). Specimens were checked every 24 h, the stones and the tissue wetted and fresh food added whenever necessary. Specimens were recorded as alive when they were capable of some movement or dead when there was no movement after being poked at. We did not attempt the potential recovery of specimens apparently moving uncoordinatedly, as all life specimens were immediately returned to the incubator to minimise disturbance of the experimental conditions. No specimen considered to be dead recovered after being retired from the experimental boxes.

Experiments were conducted in replicas of 10 specimens each, including a control kept in the same conditions at the approximate temperature of the cave (12°C for *T. ferreri*; 7°C for *T. fonti*; 6°C for *T. articollis* and 10°C for *M. infernus*; Additional file 1: Table S1), and with specimens taken directly from the stock. When the number of specimens was insufficient we used smaller replicas, and when there was no mortality the same control could be re-used for successive experiments, occasionally with less than ten individuals (Additional file 1: Table S2). We conducted three types of experiments: 1) Survival at different temperatures: treated specimens were placed directly from the stock to a set temperature (6, 20, 23 or 25°C, plus controls) and survival recorded for up to 7 days. 2) Acclimation at ramping temperatures: starting at 20°C, the temperature was raised 1°C every 48 h, until all specimens died. 3) Acclimation at fixed temperatures: specimens were acclimated for seven days at 6 or 12°C and then transferred to 23°C (Additional file 1: Table S2).

### Statistical analyses

For the comparison of the results of the short term experiments we used non-parametric statistical tests (Mann–Whitney U-test), as the dependent variable does not follow a normal distribution. We nonetheless computed parametric t-tests for comparison and to test the robustness of the results. For the long term experiments we also used non-parametric Mann–Whitney U-tests and Kruskal-Wallis to compare the survival at different temperatures, with multiple comparison tests to detect significant differences between means. Similarly, despite the non-normality of residuals we analysed the data through the use of generalized linear models (GLM) assuming a Poisson distribution and with post-hoc tests using the Bonferroni correction to identify significant differences among groups. All statistical analyses were conducted using SPSS 15.0.1 and Statistica 8.0.

## Results

### Short term experiments

When measured using a fast temperature increase (1°C/m) the average upper thermal limit (UTL) of the specimens tested was around 50°C, with no significant differences between the species in the Pyrenees (*Troglocharinus fonti*, UTL = 50.9°C, sd = 0.59) and the coastal region (*T. ferreri*, UTL = 50.7°C, sd = 0.49) (M-W U-test: U = 75.5, p = 0.06; t-test: t-value = 0.90, d.f. = 32, p = 0.37; Figure 2B and Additional file 1: Table S2A).

Using the same speed of temperature change, the average lower thermal limit (LTL) was again not significantly different between species (*T. ferreri*, LTL = −2.4°C, sd = 0.91; *T. fonti*, LTL = −2.1°C, sd 1.21; M-W U-test: U = 109, p = 0.31; t-test: t-value = 0.75, d.f. = 33; p = 0.46; Figure 2C and Additional file 1: Table S2A). The increase in temperature due to the release of the latent heat when the tissue froze at the supercooling point was around 1°C for both species, and the temperature of the specimens took on average ca. 2 minutes to match again the ambient temperature (Figure 2A and Additional file 1: Table S2A). The decrease in body temperature was perfectly linear for both species, in synchrony with the decrease of ambient temperature, with the only interruption of the release of the latent heat (Figure 2A). There was thus no indication of temperature compensation or regulation in any of the two species tested.

### Long term experiments

For the long term experiments results using non-parametric Kruskal-Wallis ANOVA (K-W), t-tests and GLM were very similar, so the latter are reported only when there were differences (see Additional file 1: Table S3 for a full account of GLM results).

#### Survival at different temperatures

**Figure 3:**
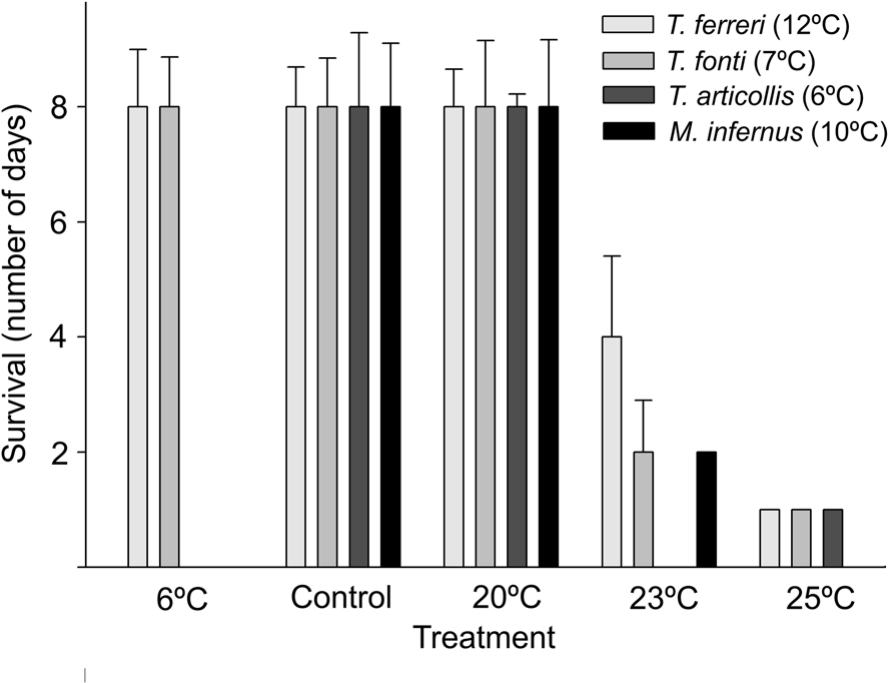
**Survival at different temperatures in the long term experiments.** Bars show the median survival, and lines the standard deviation of the average. When specimens were alive at the end of the 7-day experiment, their survival was scored as 8. In brackets, temperature of the control of the respective species.

For all species there were significant differences in the long term survival (7 days) at different temperatures (control, 6, 20, 23 and 25°C) (K-W test: H (4, N = 351) = 250.61, p < 0.0001). Survival at 6°C, controls and 20°C was in all cases almost 100% at the end of the test the 7th day. On the contrary, survival at 25°C was always less than 24 h (Figure 3 and Additional file 1: Table S2B). Survival at 23°C was more variable (Figure 3), but still overall differences among species were not significant (K-W test: H (3, N = 351) = 3.13, p = 0.37), and when compared using GLM models and a Bonferroni correction the interaction between species and treatment did not result in significant differences (p = 0.26, Additional file 1: Table S3).

#### Acclimation at ramping temperatures

There were significant differences among species in an overall comparison of the survival when acclimated with an increase of 1°C every 48 h (starting from 20°C) (K-W test: H (2, N = 25) = 11.15, p < 0.005). Pairwise differences were, however, only significant between the coastal *T. ferreri* and the most distant outgroup species tested (*M. infernus*), not between the coastal and Pyrenean species of *Troglocharinus*. Specimens of *T. ferreri* survived on average longer than those of *M. infernus*, but in any case none of the specimens survived more than 24 h at 25°C (Table 2A).

**Table 2:** **Results of the acclimation experiments**

**A)**	**n**	**T°C**	**Min.**	**Max.**	**Std.Dev.**
*T. ferreri*	9	24.3	22	25	1.1
*T. fonti*	10	23.6	21	24	1.0
*M. infernus*	6	22.2	20	23	1.2
**B)**		**No. days**	**Min.**	**Max.**	**Std.Dev.**
*T. ferreri* (6 to 23°C)	9	3.3	2	5	1.32
*T. ferreri* (12 to 23°C)*	10	4.3	2	7	1.89
*T. fonti* (6 to 23°C)	7	2.0	1	3	0.82
*T. fonti* (7 to 23°C)*	16	2.2	1	3	0.75
*T. fonti* (12 to 23°C)	8	3.7	2	5	1.16

A) Ramping temperatures (average survival temperature); B) fixed temperatures (average number of surviving days). With asterisks, control treatments.

#### Acclimation at fixed temperatures

There were no significant differences in survival at 23°C between the specimens acclimated at the control temperatures and individuals acclimated for one week at a different temperature (6°C for *T. ferreri*, M-W U-test, U = 67.5, p = 0.49; 6 and 12°C for *T. fonti*, K-W test: H (2, N = 56) = 5.92, p = 0.052) (Table 2B and Additional file 1: Table S2).

## Discussion

The homogeneity and stability of the deep subterranean environment, both at ecological and evolutionary scales, would lead to the expectation of a close match between the thermal tolerance of the species living in it and the surrounding temperatures [10,11]. This expectation was only partially fulfilled. The maximum viable temperature range for all studied species (between close to 0°C to 23°C) was much narrower than that of non subterranean insects living at the same latitude, indicating that subterranean species in this lineage do have a modified thermal biology. However, there were no significant differences between species living in caves at substantial different temperatures.

Thus, the supercooling point of the species of *Troglocharinus* living in the Pyrenees and the one living in the coastal area were the same (ca. -2.5°C), and higher than that of most species of temperate invertebrates [26,28], including other species of surface Coleoptera (see e.g. [27,29-32] for measures obtained using the same methods). The supercooling point of the studied subterranean species is probably close to the freezing temperature of hemolymph without the presence of additional osmoregulation or antifreezing substances, which has been estimated at −2.79°C for a hemolymph osmolality of 1.5 osmoles [26].

Some subterranean Leptodirini of the same Pyrenean lineage are known to live in caves with permanent snow and ice at ca. 2-3°C, such as some species of *Paratroglophyes*, or *Speonomites kryophilos* (Fresneda & Hernando) ([33]:page 571, [34]). However, due to the stability of the subterranean environment this temperature is maintained around the same value through the year, without substantial fluctuations and not reaching sub-zero temperatures. Caves at below zero temperatures are likely to be uninhabitable as water should not be available in liquid form at any time of the year. This suggests that in this lineage of cave beetles species have lost the capability to sustain low supercooling temperatures, thus avoiding the metabolic cost of synthesizing the cryoprotectants usually present in temperate insects [25,26].

We did not find differences between species in the upper thermal limits when measured in a short term ramping experiment, which in this case were similar to those found in other beetles of the same latitude (around 40-50°C, e.g. [27,29-32]), and similar to that of most eukaryotic species [28]. It seems likely that, with the exception of species living in extreme environments such as deep oceanic vents or hydrothermal sources, most short term measures of upper thermal tolerance can only be related to general biochemical or physiological constraints, and in particular to the denaturation temperature of most proteins [35]. The lack of adjustment of the short term measure of the UTL with local conditions seems to be a common pattern in terrestrial animals (e.g. [36-38]), and its ecological or evolutionary interpretation (e.g. [39]) may thus be debatable.

In sharp contrast to the estimated short term UTL, none of the species tested could survive more than 24 h at 25°C, and the survival at 23°C was also limited to 2–3 days, unlike most surface terrestrial species in temperate regions. Data on the development of some species of the same evolutionary lineage support this strict thermal limitation, with 100% egg mortality between 15-21°C [40]. The proximate cause of mortality of terrestrial animals at subcritical temperatures (i.e. pejus temperatures in the sense of [41]), and specially the role of oxygen deprivation, is controversial [42-44]. For subterranean species oxygen accessibility is complicated by the water saturated atmosphere in the cave and the increased permeability of their integument, which may blur the distinction between aquatic and terrestrial habits (as in some species of “amphibious” Leptodirini, e.g. [45,46]). In any case, it seems likely that given the narrow range of tolerated temperatures and the high mortality (100% in less than 24 h at 25°C) temperature limitation must be the result of a strong physiological constraint rather than the cumulative effect of an increased metabolic rate.

Contrary to expectations, we did not find differences in the long term thermal tolerance of the Pyrenean and the coastal species. In our tests, at 6 and 20°C the survival of the adults was in all species close to 100% at seven days. Although this time may be insufficient to assess the long term effect of subcritical (pejus) temperatures, it excludes the possibility of marked differences between the coastal and Pyrenean species of *Troglocharinus*, as 20°C is above the temperature experienced by both species in their recent evolutionary history, and 6°C is below that experienced by the coastal species since their range expansion in the early Pliocene [21] (see below and Table 1). We did not find clear signs of acclimation, specially in the two *Troglocharinus* species tested. Some results suggest the possibility of some differences in acclimation to slow ramping temperatures with respect to the most distantly related outgroup (*Macharoscelis infernus*), but in any case these differences were limited to a longer survival at intermediate temperatures, maintaining the same absolute thermal limits. Some of the developmental data available also suggest the possibility of long term acclimation, as the optimal temperatures for larval development are reported to vary depending on the temperature at which the parents were reared –but again never reaching beyond the same absolute thermal limits [40].

All evidence thus suggest that species in this lineage of Pyrenean subterranean beetles have lost some of the physiological mechanisms linked to thermal tolerance commonly present in temperate insects, and it can be hypothesized that they reflect the default limits inherited from their most closely related surface relatives in the early Miocene (Figure 1). This does not seem to be the case for other lineages of subterranean Leptodirini in different geographical areas, such as the genus *Neobathyscia* in northeast Italy. Some species in this genus have retained resistance to sub-zero temperatures even if they do not experience them in their current habitat, with the freezing temperature (also measured as the release of latent heat at the supercooling point) between −6°C and −8°C in species living at different depths in the cave [47]. They also have a better tolerance to higher temperatures [14] and have been shown to retain an HSP70 heat shock response, which also differs between species according to their current habitat [48]. It is likely that *Neobathyscia* derives from surface ancestors that diverged from the Pyrenean lineage at least since the middle Eocene [19,20], but its precise phylogenetic position and the age of the colonization of the subterranean environment are unknown. In this case, the retention of a wider tolerance can be linked to the current habitat (with the species living closer to the entrance having a wider tolerance), and possibly to a more recent origin of the strictly subterranean adaptations [47].

Other subterranean organisms have been shown to have thermal tolerances broader than the extremely narrow range of temperatures of their current environment, such as e.g. some European stygobiont Crustaceans [49,50]. Although some species were sensitive to changes of only 2°C above or below their habitat temperature, others were able to tolerate without problems changes of more than 10°C [49]. However, in this case the wider thermal tolerance was interpreted to be the result of the changes in temperature experienced during the Pleistocene [50], while in the species of *Troglocharinus* the tolerance goes beyond the temperatures experienced through their entire evolutionary history. The highest temperatures currently experienced by the species of the genus are those in the coastal area of Catalonia, with a maximum of ca. 16°C in *T. ferreri*. During the Miocene thermal optimum (16–14 Ma), with temperatures up to 3-4°C higher that at present [51], there were no Leptodirini in the coastal area, as the colonization occurred during the early Pliocene [21]. In the Pyrenean range, an increase of 3-4°C would still maintain the temperature of the caves below 20°C. Through the Pleistocene glacial periods the Pyrenean caves currently inhabited by Leptodirini suffered a decrease of 4-5°C (Table 1) but still maintained temperatures above zero, with only very few exceptions of caves assumed to have been recently colonized. Thus, a default tolerance range between temperatures close to the freezing point of water and ca. 20°C may have sufficed these beetles for the last 30 Million years, allowing them to dispense of any costly regulatory mechanism. If this were the case, some of the coastal populations of *Troglocharinus* may be now close to their physiological limit, which added to their reduced mobility and habitat confinement increases the concerns on their long term survival.

## Conclusions

In this manuscript we provide the first data on the thermal tolerance of terrestrial organisms strictly restricted to the deep subterranean environment, an ideal scenario for testing the long term adjustment of organisms to their environments. We hypothesise that these species have lost some of the physiological mechanisms related to thermal tolerance due to their likely metabolic cost in a stable environment but with severe resource restrictions. This has been possible because the "default" range (i.e. the thermal tolerance without added resistance mechanisms) was enough to allow them to survive in the constant environment of the caves through their evolutionary history. There are two main conclusions that could be inferred from our work: 1) even under what seem ideal conditions to expect a fine adjustment to temperature, other factors (in this case possibly the scarcity of resources) can override this adjustment, so that different taxa under different conditions may have similar broad tolerances; and 2) this should be a strong cautionary warning for studies trying to infer physiological tolerances from characteristics of the environment only, specially considering that in most cases organisms have many different ways to use the temporal and spatial heterogeneity of the habitat to accommodate their needs.

## Availability of supporting data

All raw data are included in the Supplementary files.

## Additional file

Additional file 1: Table S1. Temperature (T) and relative humidity (RH) at the different experimental conditions, as measured with the data loggers. **Table S2.** Results of the experiments of thermal tolerance. **Table S3.** Results of the Generalized Linear Model (GLM) analyses.
