# Supplementary material for: Lack of evolutionary adjustment to ambient temperature in highly specialized cave beetles
Source: BMC Evol Biol. 2015 Feb 4;15(1):10. doi: 10.1186/s12862-015-0288-2 (PMC4324670; doi:10.1186/s12862-015-0288-2)
Supplement: Additional file 1: Table S1. — Temperature (T) and relative humidity (RH) at the different experimental conditions, as measured with the data loggers. Table S2. Results of the experiments of thermal tolerance. Table S3. Results of the Generalized Linear Model (GLM) analyses. [file 12862_2015_288_MOESM1_ESM.docx]

**Lack of evolutionary adjustment to ambient temperature in highly specialized cave beetles**

Valeria Rizzo, David Sánchez-Fernández, Javier Fresneda, Alexandra Cieslak and Ignacio Ribera

**Additional file**

**Table S1.** Temperature (T) and relative humidity (RH) at the different experimental conditions, as measured with the data loggers.

**Table S2**. Results of the experiments of thermal tolerance.

**Table S3:** Results of the Generalized Linear Model (GLM) analyses.

**Table S1.** Temperature (TºC) and relative humidity (RH) at the different experimental conditions, as measured with the data loggers, pooled from different experiments with the same conditions. A, treatment, T and RH inside the experimental boxes in the Nüve incubator. B, controls, temperature inside the control boxes in the Liebherr cabinet. No. Obs., pooled number of registers of the data loggers for each measure (taken every 30"). For the survival and acclimation at 6ºC the conditions were the same as the control for *T. arcticollis*. These values include the alterations in T and RH produced by the observation of the boxes every 24h to count the surviving specimens, during which the data loggers continued to record the conditions inside the experimental boxes.

| **A)**  treatment | avg.T | var.T | %RH | var.RH | No. Obs. |
| --- | --- | --- | --- | --- | --- |
| 15 | 15.2 | 0.036 | 99.90 | 0.001 | 22592 |
| 20 | 20.1 | 0.025 | 99.65 | 0.480 | 260506 |
| 21 | 21.1 | 0.016 | 99.89 | 0.024 | 17271 |
| 22 | 22.1 | 0.005 | 99.92 | 0.023 | 17629 |
| 23 | 23.0 | 0.006 | 99.95 | 0.061 | 166919 |
| 24 | 24.0 | 0.007 | 99.96 | 0.069 | 8606 |
| 25 | 24.9 | 0.004 | 99.99 | 0.001 | 8769 |
|  |  |  |  |  |  |
| **B)**  controls | avg.T | var.T |  |  | No. Obs. |
| *T. arcticollis* | 5.7 | 1.117 |  |  | 71161 |
| *T. fonti* | 6.6 | 0.744 |  |  | 100243 |
| *M. infernus* | 9.7 | 0.146 |  |  | 50703 |
| *T. ferreri* | 12.0 | 0.302 |  |  | 237679 |

**Table S2.** Results of the experiments of thermal tolerance.

**A)** Short term experiments.

Lower Thermal Limit (LTL)

*Troglocharinus fonti*

Replica 1

| specimen | supercooling  point T(°C) | latent heat (LH)  increase (°C)^1^ | duration of LH  increase (s)^2^ |
| --- | --- | --- | --- |
| 1 | -1.8 | 1.8 | 140 |
| 2 | -2.4 | 1.4 | 122 |
| 3 | -1.9 | 1.2 | 145 |
| 4 | -0.4 | 1.5 | 130 |
| 5 | -2.2 | 1.4 | 110 |
| 6 | -0.5 | 1.3 | 130 |
| 7 | -3.0 | 1.9 | 100 |
| 8 | -0.7 | 1.3 | 115 |
| 9 | -2.0 | 1.5 | 135 |
| 10 | -2.1 | 1.7 | 125 |
| 11 | -6.3 | 1.7 | 128 |
| 12^3^ | - | - | - |
| average | -2.11 | 1.50 | 125.5 |
| st.dev. | 1.62 | 0.22 | 13.2 |

^1^Heat of crystallization in the sense of Lee (2010).

^2^Time it took for the temperature of the specimen to match the surrounding temperature (see Fig. 2A)

^3^The specimen unglued its elytra and its temperature could not be measured.

Replica 2

| specimen | supercooling  T(°C) | latent heat (LH)  increase (°C) | duration of LH  increase (s) |
| --- | --- | --- | --- |
| 1 | -2.7 | 1.7 | 145 |
| 2 | -2.5 | 2.0 | 130 |
| 3 | -2.0 | 1.8 | 145 |
| 4 | -1.5 | 1.8 | 127 |
| 5 | -3.4 | 2.4 | 135 |
| 6 | -1.1 | 1.5 | 105 |
| 7 | -2.9 | 1.7 | 135 |
| 8 | -1.7 | 1.7 | 112 |
| 9 | -1.4 | 1.4 | 130 |
| 10 | -3.0 | 1.6 | 122 |
| 11 | -1.7 | 1.7 | 125 |
| 12 | -1.7 | 1.4 | 140 |
| average | -2.14 | 1.71 | 129.3 |
| st.dev. | 0.74 | 0.26 | 12.2 |

*Troglocharinus ferreri*

| specimen | supercooling  T(°C) | latent heat (LH)  increase (°C) | duration of LH  increase (s) |
| --- | --- | --- | --- |
| 1 | -2.2 | 1.5 | 145 |
| 2 | -2.1 | 1.5 | 125 |
| 3 | -2.2 | 1.6 | 100 |
| 4 | -2.8 | 1.7 | 125 |
| 5 | -2.7 | 1.2 | 135 |
| 6 | -4.4 | 1.3 | 105 |
| 7 | -2.9 | 1.2 | 130 |
| 8 | -2.1 | 1.4 | 127 |
| 9 | -1.4 | 1.3 | 134 |
| 10 | -1.3 | 1.4 | 146 |
| 11 | -1.6 | 1.3 | 128 |
| 12 | -3.5 | 1.5 | 145 |
| average | -2.42 | 1.41 | 128.75 |
| stdev | 0.91 | 0.15 | 14.51 |

Upper Thermal Limit (UTL)

| specimen | *T. fonti*  Replica 1 | *T. fonti*  Replica 2 | *T. ferreri* |
| --- | --- | --- | --- |
| 1 | 51.0 | 51.0 | 50.9 |
| 2 | 50.8 | 50.8 | 50.5 |
| 3 | 51.2 | 51.2 | 50.5 |
| 4 | 51.2 | -^1^ | 50.6 |
| 5 | 51.0 | 48.6 | 51.6 |
| 6 | 51.0 | 51.0 | 50.7 |
| 7 | 51.0 | 51.0 | 51.0 |
| 8 | 51.2 | 51.6 | 49.7 |
| 9 | 51.0 | 51.6 | 51.0 |
| 10 | 51.0 | 51.0 | 51.2 |
| 11 | 51.0 | 51.0 | 50.5 |
| 12 | 51.2 | 50.0 | -^1^ |
| average | 51.05 | 50.80 | 50.75 |
| stdev | 0.12 | 0.84 | 0.49 |

^1^ The specimens unglued their elytra and their temperature could not be measured.

**B)** Long term experiments.

No. exp.: Number of the experiment

species: species used in the experiment (*Trapezodirus arcticollis*, *Troglocharius ferreri*, *Troglocharinus fonti*, *Macharoscelis infernus*)

batch: origin of the specimens used in the experiments:

| batch | cave | date | collectors |
| --- | --- | --- | --- |
| 2 | Cova St. Salvador | 15.8.2013 | A. Cieslak, J. Fresneda, I. Ribera |
| 3 | Avenc Esquerrà | 24.9.2013 | J. Comas, J. Pastor, V. Rizzo |
| 4 | Avenc Esquerrà | 9.10.2013 | J. Comas, J. Pastor, V. Rizzo |
| 5 | Cova Ormini | 21.10.2013 | J. Comas, J. Pastor, V. Rizzo |
| 6 | Gouffre du Béguet, | 12.2.2014 | C. Bourdeau, H. Brustel, O. Courtin, A. Faille, J. Fresneda, I. Ribera |

analyses: 1, survival at different temperatures; 2, acclimation at ramping temperatures; 3, acclimation at fixed temperatures. The control replicas of the experiments of survival at 25ºC were not included in the analyses, as their duration was too short.

device: I, Nüve incubator; C, Liebherr cabinet (see main text for details).

No. exx.: Number of specimens used in the experiment.

TºC: temperature of the experiment. For the average actual measured temperatures see Table S1. 20-25, acclimation experiments with a ramping temperature (1ºC/48h); X to 23, acclimation experiments at fixed temperatures: specimens were kept for a week at temperature X before being placed at 23ºC.

days from collecting: number of days since the specimens were collected

date start: starting date of the experiment

control: C, control replica; numbers: number of the experiment used as a control for the respective treatment.

length: total length of the experiment. For the analyses we considered as finished an experiment after 7 days, although some of them were maintained for longer (data not shown).

Ex. No (1 to 10): survival of each of the specimens in the experiment. The number refers to the day in which the specimen was found dead, i.e. the actual dead occurred at some point during the previous 24h. When specimens were alive at the end of the experiment they were recorded as 8, meaning that they were alive at the 8th day of the experiment. The numbers in brackets refer to the Notes below.

| No exp. | species | batch | analyses | device | No exx. | TºC | days from collecting | date start | control | length | Ex. No  1 | 2 | 3 | 4 | 5 | 6 | 7 | 8 | 9 | 10 | alive at end of experiment |
| --- | --- | --- | --- | --- | --- | --- | --- | --- | --- | --- | --- | --- | --- | --- | --- | --- | --- | --- | --- | --- | --- |
| 1 | *T. arcticollis* | 2 | 1 | C | 10 | 6 | 3 | 18.8 | C | 7 | 1 | 8 | 8 | 8 | 8 | 8 | 8 | 8 | 8 | 8 | yes |
| 2 | *T. arcticollis* | 2 | 1 | C | 7 | 6 | 11 | 26.8 | C | 7 | 8 | 8 | 8 | 8 | 8 | 8 | 8 |  |  |  | yes |
| 3 | *T. arcticollis* | 2 | 1 | I | 10 | 20 | 11 | 26.8 | 2 | 7 | 7 | 7 | 8 | 8 | 8 | 8 | 8 | 8 | 8 | 8 | yes |
| 4 | *T. arcticollis* | 2 |  | C | 3 | 6 | 25 | 9.9 | C | 1 | 2 | 2 | 2 |  |  |  |  |  |  |  | yes |
| 5 | *T. arcticollis* | 2 | 1 | I | 3 | 25 | 25 | 9.9 | 4 | 1 | 1 | 1 | 1 |  |  |  |  |  |  |  | no |
| 6 | *T. arcticollis* | 2 |  | C | 1 | 6 | 26 | 10.9 | C | 3 | 4 |  |  |  |  |  |  |  |  |  | yes |
| 7 | *T. arcticollis* | 2 | 1 | I | 2 | 25 | 26 | 10.9 | 6 | 3 | 1 | 1 |  |  |  |  |  |  |  |  | no |
| 8 | *T. arcticollis* | 2 | 1 | I | 1 | 25 | 27 | 11.9 | 6 | 3 | 1 |  |  |  |  |  |  |  |  |  | no |
| 9 | *T. ferreri* | 3 |  | C | 10 | 12 | 6 | 30.9 | C | 1 | 1 | 2 | 2 | 2 | 2 | 2 | 2 | 2 | 2 | 2 | yes |
| 10 | *T. ferreri* | 3 | 1 | I | 10 | 25 | 6 | 30.9 | 9 | 1 | 1 | 1 | 1 | 1 | 1 | 1 | 1 | 1 | 1 | 1 | no |
| 11 | *T. ferreri* | 3 | 1 | C | 10 | 12 | 7 | 1.10 | C | 7 | 1 | 8 | 8 | 8 | 8 | 8 | 8 | 8 | 8 | 8 | yes |
| 12 | *T. ferreri* | 3 | 1 | I | 10 | 20 | 7 | 1.10 | 11 | 7 | 4 | 4 | 7 | 8 | 8 | 8 | 8 | 8 | 8 | 8 | yes |
| 13 | *T. ferreri* | 4 | 1 | C | 10 | 12 | 1 | 10.10 | C | 7 | 8 | 8 | 8 | 8 | 8 | 8 | 8 | 8 | 8 | 8 | yes |
| 14 | *T. ferreri* | 4 | 1 | C | 10 | 6 | 1 | 10.10 | 13 | 7 | 7 | 8 | 8 | 8 | 8 | 8 | 8 | 8 | 8 | 8 | yes |
| 15 | *T. ferreri* | 4 | 2 | I | 10 | 20-25 | 1 | 10.10 | 13 | 7 | 5 | 6 | 8 | 8 | 8 | 8 | 8 | 8 | 8 |  | yes (1) |
| 16 | *T. ferreri* | 4 | 1 | C | 10 | 12 | 12 | 21.10 | C | 7 | 8 | 8 | 8 | 8 | 8 | 8 | 8 | 8 | 8 | 8 | yes |
| 17 | *T. ferreri* | 4 | 1-3 | I | 9 | 6 to 23 | 12 | 21.10 | 16 | 7 | 2 | 2 | 2 | 2 | 4 | 4 | 4 | 5 | 5 |  | no |
| 18 | *T. ferreri* | 4 | 1-3 | I | 10 | 12 to 23 | 12 | 21.10 | 16 | 7 | 2 | 2 | 2 | 3 | 5 | 5 | 5 | 6 | 6 | 7 | no |
| 19 | *T. ferreri* | 3&4 | 1 | C | 10 | 12 | >18 | 27.10 | C | 7 | 8 | 8 | 8 | 8 | 8 | 8 | 8 | 8 | 8 | 8 | yes |
| 20 | *T. ferreri* | 3&4 | 1 | C | 8 | 12 | >18 | 27.10 | C | 7 | 8 | 8 | 8 | 8 | 8 | 8 | 8 | 8 |  |  | yes |
| 21 | *T. ferreri* | 3&4 | 1 | C | 10 | 6 | >18 | 27.10 | 19 | 7 | 2 | 8 | 8 | 8 | 8 | 8 | 8 | 8 | 8 | 8 | yes |
| 22 | *T. ferreri* | 3&4 | 1 | C | 5/10 | 12 | >35 | 4.11 | C | 7 | 8 | 8 | 8 | 8 | 8 |  |  |  |  |  | yes |
| 23 | *T. ferreri* | 3&4 | 1 | C | 7 | 12 | >52 | 21.11 | C | 7 | 8 | 8 | 8 | 8 | 8 | 8 | 8 |  |  |  | yes |
| 24 | *T. ferreri* | 3&4 | 1 | I | 7 | 20 | >52 | 21.11 | 23 | 7 | 6 | 7 | 8 | 8 | 8 | 8 | 8 |  |  |  | yes |
| 25 | *T. ferreri* | 3&4 | 1 | C | 4 | 12 | >63 | 2.12 | C | 7 | 8 | 8 | 8 | 8 |  |  |  |  |  |  | yes (2) |
| 26 | *T. ferreri* | 3&4 | 1-3 | I | 8 | 23 | >63 | 2.12 | 25 | 7 | 1 | 2 | 2 | 3 | 4 | 4 | 4 | 7 |  |  | no |
| 27 | *T. fonti* | 5 | 1 | C | 10 | 7 | 0 | 21.10 | C | 7 | 5 | 8 | 8 | 8 | 8 | 8 | 8 | 8 | 8 | 8 | yes |
| 28 | *T. fonti* | 5 | 1 | C | 10 | 6 | 0 | 21.10 | 27 | 7 | 4 | 8 | 8 | 8 | 8 | 8 | 8 | 8 | 8 | 8 | yes |
| 29 | *T. fonti* | 5 | 1-3 | I | 10 | 23 | 1 | 22.10 | 27 | 7 | 1 | 1 | 1 | 1 | 2 | 2 | 2 | 2 | 2 | 3 | no |
| 30 | *T. fonti* | 5 | 3 | I | 7 | 6 to 23 | 8 | 29.10 | - | 6 | 1 | 1 | 2 | 2 | 2 | 3 | 3 |  |  |  | no |
| 31 | *T. fonti* | 5 | 3 | I | 9 | 7 to 23 | 8 | 29.10 | - | 6 | 1 | 1 | 2 | 2 | 2 | 2 | 3 | 3 | 3 |  | no |
| 32 | *T. fonti* | 5 | 1 | C | 10 | 7 | 21 | 12.11 | C | 7 | 8 | 8 | 8 | 8 | 8 | 8 | 8 | 8 | 8 | 8 | yes |
| 33 | *T. fonti* | 5 | 2 | I | 10 | 20-25 | 21 | 12.11 | 32 | 7 | 3 | 7 | 8 | 8 | 8 | 8 | 8 | 8 | 8 | 8 | yes |
| 34 | *T. fonti* | 5 | 1 | C | 10 | 7 | 30 | 21.11 | C | 7 | 2 | 8 | 8 | 8 | 8 | 8 | 8 | 8 | 8 | 8 | yes |
| 35 | *T. fonti* | 5 | 1 | I | 10 | 20 | 30 | 21.11 | 34 | 7 | 8 | 8 | 8 | 8 | 8 | 8 | 8 | 8 | 8 | 8 | yes |
| 36 | *T. fonti* | 5 |  | C | 10 | 12 | 32 | 23.11 | 34 | 7 | 6 | 8 | 8 | 8 | 8 | 8 | 8 | 8 | 8 | 8 | yes |
| 37 | *T. fonti* | 5 | 1 | C | 10 | 7 | 41 | 2.12 | C | 7 | 3 | 8 | 8 | 8 | 8 | 8 | 8 | 8 | 8 | 8 | yes |
| 38 | *T. fonti* | 5 | 1-3 | I | 8 | 7 to 23 | 41 | 2.12 | 37 | 7 | 1 | 2 | 2 | 2 | 3 | 3 | 3 |  |  |  | no (3) |
| 39 | *T. fonti* | 5 | 1-3 | I | 9 | 12 to 23 | 41 | 2.12 | 37 | 7 | 2 | 3 | 3 | 3 | 4 | 5 | 5 | 5 |  |  | no (4) |
| 40 | *T. fonti* | 5 |  | C | 10 | 12 | 43 | 4.12 | 37 | 7 | 1 | 8 | 8 | 8 | 8 | 8 | 8 | 8 | 8 | 8 | yes (5) |
| 41 | *T. fonti* | 5 | 1 | C | 10 | 7 | 51 | 12.12 | C | 7 | 8 | 8 | 8 | 8 | 8 | 8 | 8 | 8 | 8 | 8 | yes |
| 42 | *T. fonti* | 5 | 3 | I | 8 | 12 to 23 | 51 | 12.12 | 41 | 4 | 1 | 1 | 2 | 2 | 3 | 3 | 3 | 3 |  |  | no (6) |
| 43 | *T. fonti* | 5 | 3 | I | 9 | 7 to 23 | 51 | 12.12 | 41 | 4 | 1 | 1 | 3 | 3 | 3 | 3 | 3 | 3 | 3 |  | no |
| 44 | *T. fonti* | 5 | 1 | I | 10 | 20 | 55 | 16.12 | 41 | 7 | 2 | 2 | 3 | 4 | 8 | 8 | 8 | 8 | 8 | 8 | yes |
| 45 | *T. fonti* | 5 |  | C | 10 | 7 | 54 | 15.1 | C | 1 | 1 | 2 | 2 | 2 | 2 | 2 | 2 | 2 | 2 | 2 | yes |
| 46 | *T. fonti* | 5 | 1 | I | 6 | 25 | 85 | 15.1 | 45 | 1 | 1 | 1 | 1 | 1 | 1 | 1 |  |  |  |  | no |
| 47 | *T. fonti* | 5 | 1 | C | 4 | 7 | 86 | 16.1 | C | 7 | 6 | 8 | 8 | 8 |  |  |  |  |  |  | yes |
| 48 | *T. fonti* | 5 | 1 | I | 5 | 20 | 87 | 17.1 | 47 | 7 | 8 | 8 | 8 | 8 | 8 |  |  |  |  |  | yes |
| 49 | *M. infernus* | 6 | 1 | C | 10 | 10 | 1 | 13.2 | C | 7 | 1 | 8 | 8 | 8 | 8 | 8 | 8 | 8 | 8 | 8 | yes |
| 50 | *M. infernus* | 6 | 1 | I | 10 | 20 | 1 | 13.2 | 49 | 7 | 1 | 1 | 4 | 8 | 8 | 8 | 8 | 8 | 8 | 8 | yes |
| 51 | *M. infernus* | 6 | 1 | C | 11 | 10 | 12 | 24.2 | C | 7 | 8 | 8 | 8 | 8 | 8 | 8 | 8 | 8 | 8 | 8 | yes |
| 52 | *M. infernus* | 6 | 1 | I | 10 | 20 | 12 | 24.2 | 51 | 7 | 6 | 6 | 8 | 8 | 8 | 8 | 8 | 8 | 8 | 8 | yes |
| 53 | *M. infernus* | 6 | 1 | C | 5 | 10 | 26 | 10.3 | C | 7 | 8 | 8 | 8 | 8 | 8 | 8 |  |  |  |  | yes |
| 54 | *M. infernus* | 6 | 2 | I | 6 | 20-25 | 26 | 10.3 | 52 | 7 | 1 | 4 | 5 | 6 | 7 | 7 |  |  |  |  | no |
| 55 | *M. infernus* | 6 |  | C | 3 | 10 | 33 | 17.3 | C | 2 | 3 | 3 | 3 |  |  |  |  |  |  |  | yes (7) |
| 56 | *M. infernus* | 6 | 1 | I | 4 | 23 | 33 | 17.3 | 55 | 2 | 2 | 2 | 2 | 2 |  |  |  |  |  |  | no (8) |

**Notes:**

(1) 1 accidental death 11.10

(2) previously at 20ºC (experiment 24)

(3) 1 ex. missing 3.12

(4) 1 ex. missing 3.12

(5) 1 accidental death 10.12

(6) 1 accidental death 15.12

(7) in two batches: 1st 2 exx 17.3, 2nd 1 ex. 19.3

(8) in three batches: 1st 2 exx 17.3, 2nd 1 ex. 19.3; 3rd 1 ex. 25.3 without control

- **Table S3.** Results of the Generalized Linear Model (GLM) analyses.

**A)** Survival at different temperatures

| Source | - Wald Chi-Square | - df | - Sig. |
| --- | --- | --- | --- |
| - intercept | - 546.883 | - 1 | - <0.0001 |
| - species | - 2.226 | - 3 | - 0.53 |
| - treatment | - 151.986 | - 4 | - <0.0001 |
| - treatment x species | - 10.023 | - 8 | - 0.26 |

**B)** Acclimation at ramping temperatures.

| - Source | - Type III Sum of Squares | - df | - Mean Square | - F | - Sig. |
| --- | --- | --- | --- | --- | --- |
| - corrected model | - 17.007^a^ | - 2 | - 8.503 | - 7.414 | - <0.005 |
| - intercept | - 13007.674 | - 1 | - 13007.674 | - 11340.904 | - <0.0001 |
| - species | - 17.007 | - 2 | - 8.503 | - 7.414 | - <0.005 |
| - error | - 25.233 | - 22 | 1.147 |  |  |
| - Total | - 13872.000 | - 25 |  |  |  |
| - corrected Total | - 42.240 | - 24 |  |  |  |
| - ^a^ R Squared = 0.403 (Adjusted R Squared = 0.348) | | | | | |

| Posthoc (Bonferroni) | | | | | | | |
| --- | --- | --- | --- | --- | --- | --- | --- |
| - (I) species | - (J) species | mean (I-J) | - Std. Error | - Sig. | - 95% C.I. | |  |
| - *T. ferreri* | - *T. fonti* | - 0.73 | - 0.49 | - 0.45 | - -0.54 | - 2.01 |  |
|  | *M. infernus* | - 2.17^*^ | - 0.56 | - <0.005 | - 0.70 | - 3.63 |  |
| - *T. fonti* | - *T. ferreri* | - -0.73 | 0.49 | - 0.45 | - -2.01 | 0.54 |  |
|  | - *M. infernus* | - 1.43^*^ | - 0.55 | - <0.05 | - 0.00 | - 2.87 |  |
| - *M. infernus* | - *T. ferreri* | - -2.17^*^ | - 0.56 | - <0.005 | - -3.63 | - -0.70 |  |
|  | - *T. fonti* | - -1.43^*^ | - 0.55 | - <0.05 | - -2.87 | - 0.00 |  |

**C)** Acclimation at fixed temperatures.

| *T. ferreri* | | | |
| --- | --- | --- | --- |
| - Source | - Type III | | |
|  | - Wald Chi-Square | - df | - Sig. |
| - intercept | 375.246 | - 1 | - <0.0001 |
| - treatment | - 0.071 | - 1 | - 0.79 |

| *T. fonti* | | | |
| --- | --- | --- | --- |
| - Source | - Type III | | |
|  | - Wald Chi-Square | - df | Sig. |
| - intercept | - 63.098 | - 1 | - <0.0001 |
| treatment | - 3.304 | - 2 | - 0.19 |
